# Site Fidelity in Space Use by Spider Monkeys (*Ateles geoffroyi*) in the Yucatan Peninsula, Mexico

**DOI:** 10.1371/journal.pone.0062813

**Published:** 2013-05-13

**Authors:** Gabriel Ramos-Fernandez, Sandra E. Smith Aguilar, Colleen M. Schaffner, Laura G. Vick, Filippo Aureli

**Affiliations:** 1 Centro Interdisciplinario de Investigación para el Desarrollo Integral Regional, Unidad Oaxaca, Instituto Politécnico Nacional, Santa Cruz Xoxocotlán, Oaxaca, México; 2 Instituto de Neuroetología, Universidad Veracruzana, Xalapa, Veracruz, México; 3 Department of Psychology, University of Chester, Chester, United Kingdom; 4 Department of Anthropology, William Peace University, Raleigh, North Carolina, United States of America; 5 Research Centre in Evolutionary Anthropology and Palaeoecology, School of Natural Sciences and Psychology, Liverpool John Moores University, Liverpool, United Kingdom; Texas A&M University, United States of America

## Abstract

Animal home ranges may vary little in their size and location in the short term but nevertheless show more variability in the long term. We evaluated the degree of site fidelity of two groups of spider monkeys (*Ateles geoffroyi*) over a 10- and 13-year period, respectively, in the northeastern Yucatan peninsula, Mexico. We used the Local Convex Hull method to estimate yearly home ranges and core areas (defined as the 60% probability contour) for the two groups. Home ranges varied from 7.7 to 49.6 ha and core areas varied from 3.1 to 9.2 ha. We evaluated the degree of site fidelity by quantifying the number of years in which different areas were used as either home ranges or core areas. Large tracts were used only as home ranges and only for a few years, whereas small areas were used as either core area or home range for the duration of the study. The sum of the yearly core areas coincided partially with the yearly home ranges, indicating that home ranges contain areas used intermittently. Home ranges, and especially core areas, contained a higher proportion of mature forest than the larger study site as a whole. Across years and only in one group, the size of core areas was positively correlated with the proportion of adult males in the group, while the size of home ranges was positively correlated with both the proportion of males and the number of tree species included in the diet. Our findings suggest that spider monkey home ranges are the result of a combination of long-term site fidelity and year-to-year use variation to enable exploration of new resources.

## Introduction

A notable feature of animal movements is the existence of home ranges (HR), or areas where animals regularly travel to search for food [1]. The existence of site fidelity, or a stable use of space that varies little over time, is primarily due to the importance of familiarity with a known area, which is particularly advantageous for animals living in heterogeneous environments [2]. Site fidelity could also be related to the active defense of the HR or a portion of it, in the case of territorial animals [1], [3].

Within their HR, animals tend to concentrate their activities within particular regions or core areas (CA). These may contain particular habitat features, such as preferred food resources, sleeping sites or refuges [3], as has been demonstrated for several species (e.g., Siberian flying squirrels, *Pteromis volans* [4]; West Indian manatee, *Trichechus manatus*, [5]; grey mouse lemurs, *Microcebus murinus* [6]; California sheephead, *Semicossyphus pulcher* [7]; forest elephants, *Loxodonta africana cyclotis* [8]). Spider monkeys (*Ateles* spp.) also spend more time within a CA of their HR [9]–[12]. Species in this genus prefer older vegetation types and upper canopy levels [10]. In heterogeneous environments, spider monkeys may be using the oldest vegetation types more than others because they contain a greater density of available food trees [12].

While the HR of an animal may show some degree of stability, its shape and size can also change in response to environmental variation [13]. In spider monkeys, variation in the size and shape of the HR across years has been attributed to the variability in the distribution and abundance of food resources; thus HRs and, particularly, CAs should vary depending on the location of the resources used in any given season or year [14]. Indeed, Asensio et al. [11] showed that CAs vary in size and location more than HRs across a 4-year period, and that the superposition of yearly CAs largely coincides with the HR used in any given year. This pattern may be important for territorial species, such as spider monkeys, which defend a stable HR, as it contains not only the current, but also the future CAs [11].

Site fidelity could partly be due to the presence of important vegetation types and food resources, as has been shown in several species (bobolinks, *Dolichonyx oryzivorus* [15]; Eurasian red squirrel, *Sciurus vulgaris* [16]; amberwing dragonfly, *Perithemis tenera* [17]; chipping sparrows, *Spizella passerina* [18]). In a one-year study of a group of black-faced black spider monkeys (*Ateles chamek*), seasonal fruit availability was shown to have a strong influence on the size and general location of CAs, producing little overlap between seasonal CAs [19]. Likewise, the density of fruiting trees was higher in core than in non-CAs for a group of *A. geoffroyi* over a four-year period [12] and, in *A. belzebuth*, a strong degree of overlap in individual CAs occurred across years, which was ascribed to monkeys visiting the same locations to utilize key resources [20]. Although little is known about the mechanisms leading individuals in a group to restrict their movements to a confined area [2], Jetz et al.’s [21] theoretical model can be used to develop some general predictions about the relationship between the size of an area and the presence of important resources. Considering factors such as body mass, metabolic rate and the influence of neighbors, this model [21] predicts that as the resources per unit area increase, the size of the HR should decrease. Similarly, when the diet is more concentrated, the area used by a group would be expected to be smaller.

Social factors may also be important sources of long-term variation in HR size and location. For example, differences in the HR overlap of brown bears (*Ursus arctos*) may reflect changes in territorial behavior, which in turn result from changes in food abundance and predictability [22]. Mountain gorillas (*Gorilla beringei beringei*) shift their HR as a result of male mating competition [23]. In spider monkeys, it is the males that are involved in the patrolling of the HR, spending more time near the boundaries than females [9]. Thus, their capacity to patrol may affect both the size and location of the HR. As evidence of this, Wallace [10] found that the extent of ‘risky’ boundaries, which neighbor other groups’ ranges, was positively associated with the number of males in the group (cf. [20]).

In our study we used a long-term dataset from two groups of spider monkeys (*Ateles geoffroyi*) in Punta Laguna, in the northeastern Yucatan peninsula, Mexico, to evaluate the degree of site fidelity in HRs and CAs over a 10- and 13-year period. First, we estimated yearly HRs and CAs through the Local Convex Hull (LoCoH) method. Second, we quantified the degree of site fidelity by the number of years in which different areas were used as HR and CA. Finally, we evaluated the following predictions regarding the different factors that could explain variation in HR and CA size and location: a) HRs and CAs should contain a higher proportion of mature vegetation than what is available in the habitat overall; b) as the proportion of males in a group increases, there should be an increase in the size of HR; c) HRs and CAs should be larger when there is a higher diversity in the diet.

## Methods

### Ethical Statement

All observations were carried out in accordance with the current laws of Mexico. Permission to carry out the survey was granted by permit DGVS-01241.

### Study Site and Subjects

Two groups of spider monkeys (Eastern and Western) have been studied since June 1996 near the Punta Laguna lake (20°38′N, 87°37′W) in the *Otoch Ma’ax Yetel Kooh* protected area (also known as Punta Laguna [24]). Regional climate is characterized by two seasons: a rainy season from May through November and a dry season from December through April. Mean annual temperature is 24.3°C, and mean annual precipitation is 1265 mm [25]. The dominant vegetation in the region is medium semi-evergreen forest with different successional stages [26]. Slash-and-burn agriculture has traditionally been the main land use in the area and has produced a land cover mosaic of managed and unmanaged vegetation that includes recently abandoned plots as well as forest older than 50 years, interspersed with agricultural fields, vegetation corridors, water bodies and house gardens [26]. This heterogeneous land cover is commonly found in the humid tropics of Mexico, especially where cattle ranching is not a widespread activity [27].

Group size and composition varied over the study years, with the number of adult females ranging from 6–11 in the Eastern (E) group and from 13–22 in the Western (W) group; and the number of adult males ranging from 1–6 in the E group and from 7–13 in the W group. The duration of observations also varied over the study period, ranging yearly from 66 to 1048 hours in the E group (average: 602±326 SD) and from 50 to 590 hours in the W group (average: 274±198 SD). After 2006, the W group was observed less frequently, probably due to their ranging more widely as a result of the damage produced by two hurricanes in 2005 [28]. Therefore, data for this group only includes the period 1997–2006 (10 years).

Data were collected by trained field assistants. Due to their high degree of fission-fusion dynamics, spider monkey groups are split in different subgroups which vary in size and composition [29]. Therefore, data were collected following subgroups from the E and W groups. Subgroups were defined by a chain rule of 30 m, i.e. individuals were considered part of the same subgroup when they were within 30 m of at least one subgroup member [30]. Upon encountering a spider monkey subgroup, instantaneous scan samples were taken every 20 minutes until the observation period ended or contact with the subgroup was lost. During these scan samples the assistants recorded the identity of all subgroup members along with the food items and species eaten. The location of the subgroup was determined with respect to known landmarks such as trees and forks in the trail system or by using a GPS unit (Garmin eTrex, ±7 m accuracy, on average) whenever there was no landmark nearby. A map containing all landmarks was created using GPS locations superimposed on map of the trail system generated using measuring tape and compass [31].

Vegetation maps were generated using a 1999 panchromatic IRS image (6 m ground resolution) and a 2003 SPOT image (5 m ground resolution). These two images were interpreted and digitized, with land use categories and vegetation succession stages established visually for the whole protected area (5367 ha); thus, no automatic classification was required. Categorization was aided by ground verifications in which local inhabitants participated, arriving to the following land use and vegetation successional stage categories: agricultural units (*milpas*, 0–1 year), four forest succession stages (2–7, 8–15, 16–29, and 30–50 years), old-growth forest (>50 years), bodies of water and other vegetation covers (e.g. swamp grassland). The accuracy of the classification was evaluated by sampling a total of 128 sites spread throughout the protected area between 2003 and 2006, with the successional stage category assigned correctly in 90% of the sites. (see [26] for more details about the vegetation maps).

### Home Range and Core Area Analysis

A total of 32,113 20-minute instantaneous scans were analyzed for the study period, with a mean (±SD) of 1,235 (±984) scans per year: 1806 (±979) for the E group and 664 (±596) for the W group. The locations of all scan samples corresponding to a given year for each group were used to estimate yearly utilization distributions (UDs) in ArcGIS 9.2 [32]. Given the regularity in the 20-minute interval between observations in our study, we chose to prioritize the information content of the whole dataset, where repeated sequential points are an indication of intensity of use, rather than use a subset to reduce pseudoreplication [33]–[35].

We used a recently developed method, the Local Convex Hull (LoCoH; [36]), to construct UDs from the union of convex polygons associated with each observation point and their *k* closest neighbors. Thus, each point has an associated polygon which, together with all other polygons, forms “sub-layers” that comprise a certain proportion of the points in the sample and which are translated into distribution isopleths. In this manner, a sub-layer which comprises 20% of the data represents an isopleth of 20%. The advantage of this method is the capacity to identify limits or real barriers to the spatial distribution of animals because it depends on actual data, converging toward a real distribution with increasing sample size [37].

For each group and year combination, we obtained the UDs using the *LoCoH for ArcGIS toolbox* [38]. In preliminary analyses we also generated UDs using the Kernel Density Estimation (KDE; [39]). A qualitative examination of the resulting polygons showed that the KDE estimations were less accurate (Figure S1 in Supporting Information). LoCoH has also proven better for analyzing datasets in which the distribution of the points is not uniform, such as ours, which are reportedly quite challenging for the KDE method [36], [40], [41].

Although there is no biologically supported method to define a given probability contour as the HR, we adopted Laver & Kelly’s [42] recommendation that, because of its recurrent use, the 95% contour should be defined as the HR for comparison and consistency between studies. In the case of the CA we followed Powell’s [3] proposal to objectively define it as the probability contour corresponding to the inflection point in the plot of the isopleth values from 10% to 95% against the proportion that each value represents out of the total HR. The plotted points were then fitted to an exponential regression (*y* = *e^bx^*) forced through the origin, where *y* is the percent of the HR and *x* is the isopleth value. The fitted function was used to find the value of *y* where the slope of the tangent *b* equals 1 (i.e. the inflection point). Data from both groups combined and separately produced functions with inflection points lying around an *x* value of 56, prompting our decision to use the 60% probability contour as the CA for all our analyses.

### Parameter Selection for Home Range Analysis

We used the adaptive LoCoH modality, in which individual hulls are constructed using all the neighboring points within a sphere around a center point [42]. The size of each sphere varies in a way that the sum of the distances between the center and neighboring points adds up to less than or equal to a pre-established parameter *a* [42]. This variant allows for the hulls in clumped areas to be constructed with an increasing number of neighboring points. The value of *a* was established using the “minimum spurious hole covering rule” that involves reducing the value of *a* until the area calculated avoids non-existing holes in the distribution [36]. This criterion requires sufficient *a priori* knowledge on the distribution area and ecology of the species, to allow the recognition of biologically significant limits or barriers in the HR. In our case, we used the lake to “calibrate” the value of *a* by minimizing the areas from the estimated HRs that overlapped with the water (where the monkeys were never observed), but preventing the exclusion of areas of vegetation that were known to be used by the monkey groups. We thereby reduced the occupation of unlikely locations and avoided fragmented representations of a distribution known to be continuous. Given that *a* depends on the distribution of the location points in each year [42], we used different values of *a* for each group/year combination.

### Site Fidelity Analysis

**Figure 1:**
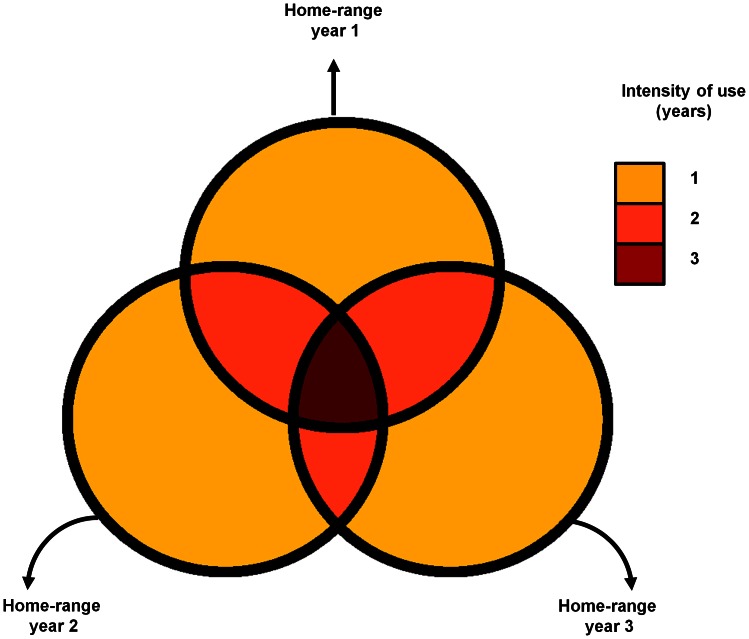
Schematic representation of three hypothetical yearly HRs (circles) that overlap partially. The color scale is the intensity of use in our site fidelity analysis and indicates the number of layers that intersect in a given region, which in this simple example can go from 1 to 3. For example, areas ranked with 2 include all portions that were used in two different years irrespectively of which years and whether they were consecutive; thus they include the overlap between years 1 and 2, 1 and 3 and 2 and 3.

In order to evaluate the degree of site fidelity of HR and CA location over the long term, we used an analysis based on the recurrent use of particular areas. First, we used ArcGIS to determine the area that included all the yearly HRs or CAs throughout the study, which we refer to as the *long-term HR* or *long-term CA.* Then, we determined the number of years on which different areas within the long-term HR or CA were used. For this, we overlaid all yearly polygons and distinguished areas covered by different numbers of yearly layers (from 1 to 13 years in the E group and from 1 to 10 years in the W group), independently of which particular year was involved (Figure 1). We refer to these areas as the *1–13 year HR* (or CA) *intersections*. For example, the 6-year HR intersection includes the area used repeatedly as HR for 6 years, independently of which particular years were involved. By defining site fidelity in this way, we focused not on the overlap pattern between pairs of consecutive yearly layers [43] but on the overall pattern of intersection between many layers corresponding to different years.

### Statistical Analysis

Yearly HRs and CAs were compared between groups using a Wilcoxon rank sum test. The proportion of different vegetation types included in the total HR and CA polygons for each group was compared to the expected proportion based on the distribution of different vegetation types in the protected area as a whole (5367 ha) using a Chi-square test with p-values computed by Monte Carlo simulation in R [44]. For this analysis, we used the successional stage categories and an “other” category, which included small areas of swamp grassland and bodies of water that were included in the HR polygons (see Results). In order to explore the association between the size of the HR or CA variables related to the size of groups and the diet, we ran a stepwise multiple regression with the yearly size of polygons (HR or CA) as dependent variables. The predictor variables were the yearly values of group size, the proportion of adult males in the group, number of species included in the diet and the proportion of the diet composed by the top five species. We selected the best regression model using the “stepAIC” procedure in R [44], which is a combination of backward elimination and forward selection. Starting with an initial model including all four variables, this procedure drops variables one at a time but, with every step, attempts to re-introduce some of the variables that were rejected in previous iterations of elimination. The final model is the one with the lowest overall Akaike Information Criterion corrected for small sample size (AICc; [45]).

## Results

**Figure 2:**
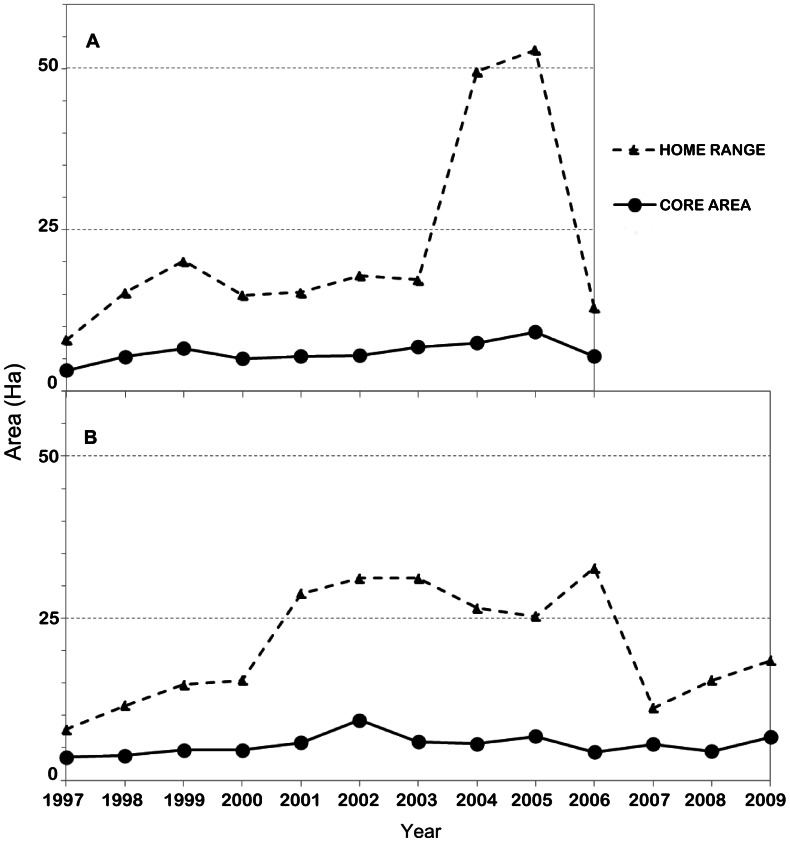
HR (dotted lines) and CA (continuous lines) estimates for the two study groups (A: W group; B: E group) during 10 and 13 years of study, respectively.

**Figure 3:**
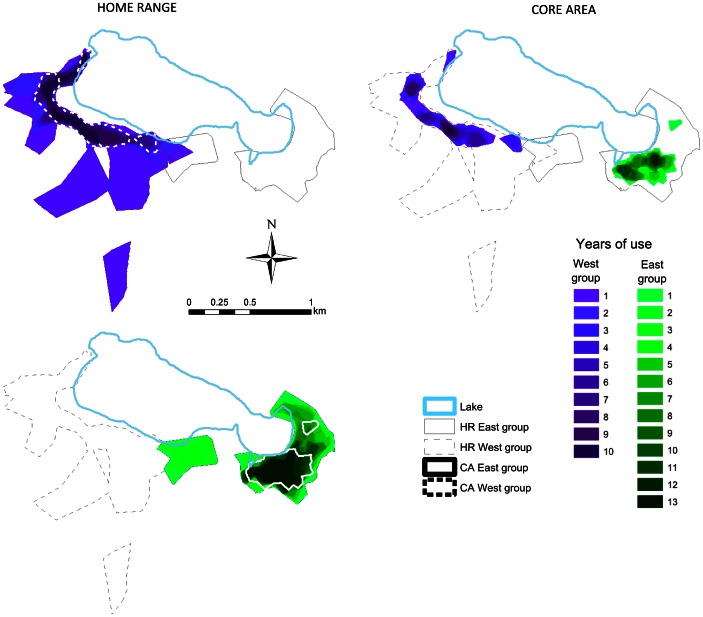
Map of the intersection of yearly HRs and CAs for the two study groups. The different colors represent the 1–13 year intersections for HR or CAs. We illustrate the partial coincidence of long-term CAs with the 13-year HR intersections by placing the long-term CA contours in the HR map of each group (thick lines: continuous, E group; dotted, W group). Long-term HRs are also noted as thin gray lines (continuous, E group; dotted, W group). Note the areas where the HRs of both groups intersect.

Figure 2 shows the wide variation existing in the HR and CA sizes for both groups. However, the estimations showed no significant differences between the two groups (Table 1). Like their size, the general location of the HR and CA was also variable (see all polygons in Figure S1 in Supporting Information). Figure 3 shows the result of overlaying the HRs (or CAs) for different years, highlighting those areas used repeatedly for different numbers of years (as explained in Figure 1). Areas closer to the Punta Laguna lake were used during more years than areas farther from the lake. Also, in both groups the long-term CA coincides to a certain extent with areas used repeatedly as HR. Even if variable across years, the HR normally includes the sum of all yearly CAs. Figure 3 also shows some small areas where both groups overlap in their HR, although these were used only for a few years by each group. Close inspection of the yearly HR (Figure S1) reveals that groups alternate the use of this region.

**Table 1:** Summary of CA and HR variation across years and between-group comparison.

Polygon	E group mean±SD (min-max)	W group mean±SD (min-max)	Wilcoxon rank sum W	P
CA	5.47±1.52 ha (3.57–9.26)	5.95±1.63 ha (3.13–9.13)	76	0.49
HR	18.73±6.55 ha (7.86–28.8)	21.63±14.08 ha (7.75–49.65)	60	0.76

**Figure 4:**
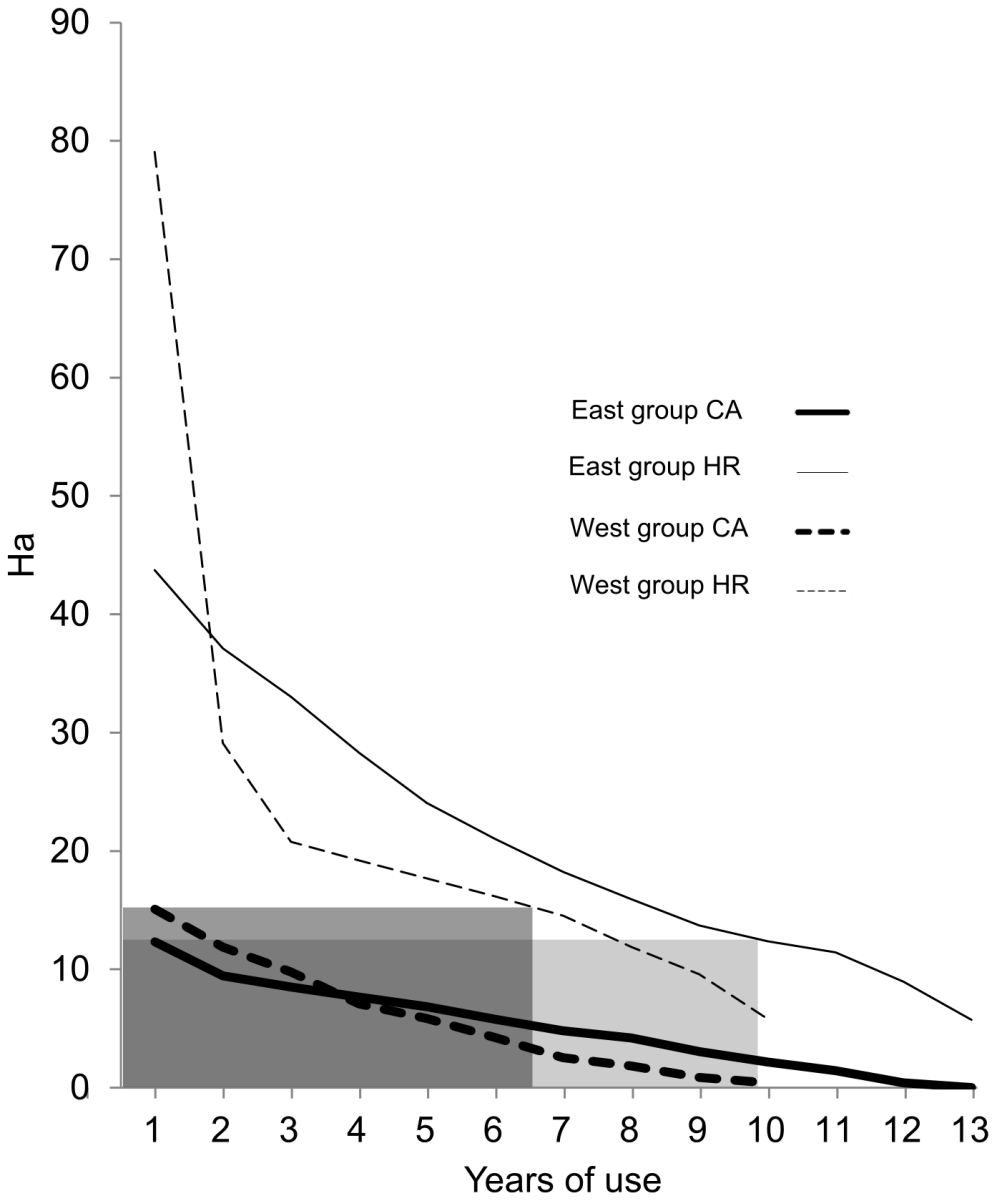
Areas repeatedly used as CA (thick lines) and HR (thin lines) by each group (group E, continuous lines; group W, dotted lines) for different numbers of years. The horizontal axis corresponds to the number of years of repeated use, with areas used repeatedly for a small number of years including areas used for more years. The vertical axis shows the size of the area for a given number of years of repeated use. The grey rectangles show the correspondence between the long-term CA and the most similar-sized HR intersection. Thus, the W group’s long-term CA corresponds to the 6–7 year HR intersections, while the E group’s long-term CA corresponds to the 10-year HR intersection.

In order to quantitatively analyze the degree of site fidelity of the HRs and CAs over time we calculated the size of the different shades of polygons shown in Figure 3, representing areas used repeatedly for different numbers of years (Figure 4). A constant use of space, in which the same exact area was used in all years, would show a horizontal line in this graph. In contrast, a highly unstable use of space, in which there was a low degree of site fidelity, would show very high values in the left of the graph and very low values for areas used repeatedly for more than a few years. The data reveal an intermediate situation, with high values for low numbers of years and a steep decrease, particularly for HRs. Neither HRs nor CAs reached an asymptote, which would correspond to a particularly important area that was always used. However, the curves do decrease more slowly for larger numbers of years of repeated use, indicating some areas that were used repeatedly, but not for the whole duration of the study. These areas can be seen in the darker tones in Figure 3.

The curves in Figure 4 quantify the pattern that is apparent in Figure 3: the larger, lighter colored areas are used only for a small number of years, while the smaller, darker areas are used for a larger number of years. CA size also decreased depending on the number of years, but did so less steeply than the size of HRs. The different shapes of these curves imply that HRs are less stable than CAs over time. The same pattern can be found in both groups. In all cases, the 13-year HR intersections are somewhat smaller than the long-term CA. The grey rectangles in Figure 4 show the HR intersection layer most closely resembling the long-term CA: the 6–7 year HR intersection for the W group and the 10 year intersection for the E group.

**Figure 5:**
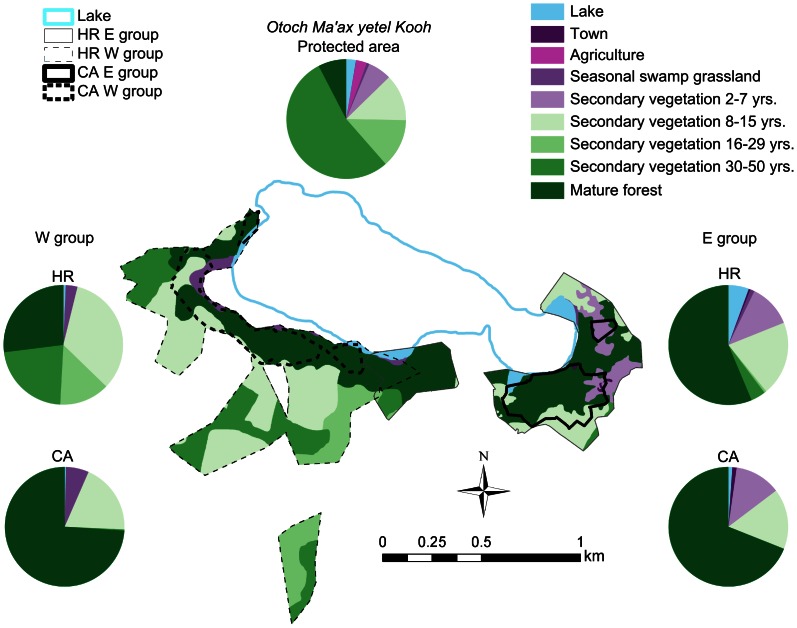
Map of vegetation types included in the HRs (thin lines) and CAs (thick lines), considering areas used for at least one year (see long-term contours in Figure 3) by the two study groups. Pie charts show the proportion of different vegetation types lying within the 5367 ha of the protected area as a whole (middle chart) and in the HR and CAs.

Figure 5 shows the vegetation composition of the long-term HR and CA. For comparison, the proportion of the different vegetation types available in the entire protected area is shown (5367 ha. in total: see Methods). It is clear that in both groups the proportion of different vegetation types in the HRs and in the CAs is different from that in the whole protected area (E group HR: χ^2^ = 166.16, P = 0.0005; W group HR: χ^2^ = 86.62, P = 0.0005; E group CA: χ^2^ = 70.48, P = 0.0005; W group CA: χ^2^ = 98.73, P = 0.0005). The proportion of mature forest was significantly higher in the CAs than in the HRs in the W group (χ^2^ = 16.89, P = 0.0005) but not in the E group (χ^2^ = 0.81, P = 0.3978).

Multiple regressions between the size of the HRs and CAs and the size of groups and diversity of diet showed significant results only for the E group (Table 2). Particularly, in the case of the CA, the proportion of males was retained as the only significant predictor (R^2^ = 0.41, P = 0.02). In other words, the proportion of males in the E group was positively associated with a larger CA across years. In the case of the HR, both the proportion of males and the total number of species in the diet were retained as significant predictors (R^2^ = 0.68, P = 0.04). Thus, the larger the proportion of males in the group and the more food species consumed by the E group in a given year, the larger the HR. None of the regressions for the W group showed significant results (Table 2).

**Table 2:** Summary of multiple regression results.

Group and polygon	Predictor variables included in initial and final regression model	R^2^	P	AICc
W group CA	Initial: group size, prop.males, diet.spp, diet5top	0.063	0.98	NA
W group HR	Initial: group size, prop.males, diet.spp, diet5top	0.358	0.62	72.41
	Final: diet5top (−)	0.23	0.16	54.94
E group CA	Initial: group size, prop.males, diet.spp, diet5top	0.44	0.27	21.01
	Final: prop.males (+)	0.41	0.02*	8.3
E group HR	Initial: group size, prop.males, diet.spp, diet5top	0.68	0.04*	51.53
	Final: prop.males (+), diet.spp (+)	0.68	0.04*	41.92

Predictor variables are group size, proportion of males (prop.males), number of species included in annual diet (diet.spp) and proportion of annual diet comprised by the top 5 species (diet5top). The sign in parenthesis after the predictor variable in the final model indicates the direction of the effect. Asterisks denote statistical significance when P<0.05. NA: not applicable as the final model included the intercept only.

## Discussion

Our results show a strong degree of site fidelity in space use by two groups of spider monkeys over a long-term period, both at the HR and CA levels. HRs and CAs varied in size over time, but they tended to coincide in the same general locations. HRs were more variable than CAs, and the area used repeatedly as HR coincided to a large extent with the area containing all CAs (i.e. the long-term CA).

Both study groups tended to concentrate their activity in areas closest to the lake, which consist mainly of mature, well-preserved forest. CAs, in particular, contained much higher proportions of mature forest than the study area as a whole, supporting Prediction a. This vegetation type contains the largest trees, particularly of those species that spider monkeys consume more often [46]. For example, *Brosimum alicastrum*, one of the main species in their diet, is 10 times more abundant in mature forest than in the successional vegetation areas that are also part of the groups’ HRs [46]. It is possible that spider monkeys are forced to use larger areas when the mature forest does not provide enough food. Our results on the diversity of diet support this interpretation: the E group’s HR was larger when the number of species included in the yearly diet was larger. This result is what we predicted (Prediction c) on the basis of the theoretical model by Jetz et al. [21]. Obviously, the analyses carried out in this study do not allow us to establish a causal relationship between the two variables. Hypothetically, both the size and location of CAs could be influenced by a third, unknown factor, and we would simply observe monkeys feeding on whatever they find in those areas.

As predicted (Prediction b), in one of our study groups we found a positive association between the proportion of adult males and the size of the group’s CA and HR across years. Spider monkey males have been reported to “patrol” the boundaries of their HR, forming larger subgroups in areas that neighbor other groups’ ranges [10]. It is possible that, regardless of the number of females, a group with a higher proportion of males can form larger patrolling subgroups and therefore cover a larger area, extending the group’s CA and HR temporarily. Whether this expansion would be at the expense of the neighboring group’s HR is not clear, but there are apparently reciprocal changes in the size and location of the HR of one group relative to the other in different years, supporting possible expansion and reduction of the HR along the boundaries of the two neighboring groups (Figure 2 and Figure S1 in Supporting Information). Future studies should attempt to uncover the relationship between patrolling behavior, the size of these patrolling subgroups and the size of HRs.

Site fidelity (and HR behavior, in general) could be the result of animals using memory-based movements to return to previously visited sites [47], [48]. There is some evidence that HRs can be expected if animals move randomly in the environment and keep an updated record of the fruiting status of preferred spots [49]. If the environment also presents changes in the local abundance of resources, then a combination of random exploration with memory-based processes could lead to shifts in the size and location of HRs [2]. We have evidence that the spider monkeys at our study site use such a combined strategy [50], [51]. It is possible that memory-based processes reinforce the use of a known area, while random explorations could help monkeys find new sources of fruit. These random explorations are reflected in areas of the HR that are used for only a few years out of the total duration of the study (Figure 3 and Figure S1). If successful, these random explorations can be reinforced by memory and thus be incorporated into the long-term patterns of space use.

We have presented results based on the LoCoH, a relatively new methodology for estimating HR and CA. Although the comparison with the KDE revealed similar patterns in the temporal variation as well as in the degree of site fidelity, the absolute values of the size of the estimated contours are very different, with LoCoH contours being about half the size as KDE (Figure S1). This agrees with the results of van Beest et al. [52], which show ample differences in absolute sizes of HRs using both methods, although qualitatively similar patterns. It is known that the LoCoH method is better than the KDE at handling irregular shapes, excluding areas without necessarily fragmenting the contours [36], [53]. In our study, the first, most obvious source of the difference is that the KDE estimation includes some areas of swamp grassland or even water that surrounds the areas of forest that the monkeys use. Given that monkeys were never actually observed in these land covers, the KDE overestimated the areas used by spider monkeys precisely where there are sharp turns in the shape of the estimated polygons, as in the southwestern corner of the lake for the W group’s range (Figure S1).

Our yearly estimations for the HRs are several-fold smaller than those reviewed by Wallace [14] and those more recently reported by Asensio et al. [11]. One possible explanation is that the mature forest in Punta Laguna, particularly around the lake, is a hyper-abundant foraging environment for spider monkeys. A very high density of *Brosimum alicastrum*, one of the preferred species in the monkeys’ diet, is consistent with this characterization [46]. However, the tree diversity, which is comparable to other sites in the northeastern Yucatan peninsula [54], is not high compared to other, hyper-diverse sites [55]. Given the large difference we found between the 1-year and the 13-year HR intersections, which in turn is due to important variations in the size and location in the HR for each year, it is possible that the most significant figures to report as the “typical” HR for spider monkeys in this study site would be the long-term HR (i.e. the area including all the yearly HRs), which would cover 44 ha in the E group and 84 ha in the W group, figures that are closer to those reviewed by Wallace [14].

We have employed a method to quantify the degree of site fidelity that, to our knowledge, has not been used before. Site fidelity is often measured by the degree of overlap between pairs of polygons corresponding to consecutive time intervals (e.g. [43]). Here we have employed the intersection of all yearly layers and spatially represented and quantified the areas used as HRs and CAs for different numbers of years. This allowed us to identify areas that are used throughout the whole study or portions of it, and to examine the relationship between the area most frequently used as HR (the 10- or 13-year intersection) and the superposition of all CAs (the long-term CA, or what Asensio et al. [11] labeled the “super-core area”). We found that the longer term HR intersection (10 or 13 years, depending on the group) is around 50–70% smaller than the long-term CA, implying that the monkeys in our study groups did not use a HR that included “all future core areas” as Asensio et al. [11] suggested based on their 4-year findings. HRs are more variable than would be expected based on the sole existence of (present and future) CAs within them. One possible reason is that HRs must always include areas that are used by monkeys to explore new sources of fruit, as suggested above.

Our findings have important implications for planning conservation strategies of spider monkeys and their habitat. First, the year-to-year variation in size and location of the HRs, as well as the difference between the yearly figures and the long-term HR size, imply that short-term data (i.e. data collected over only one year) are insufficient for inferring space use patterns in a given group or population. Therefore, management and conservation decisions should take long-term variation in space use into account. Second, although conservationists commonly assume a uniform density of individuals, for any target species, throughout an area with similar vegetation [56], the fact that HRs and CAs contain higher proportions of mature forest than the area as a whole is important for estimating the minimum area containing viable populations. However, one cannot conclude that mature forest is the only vegetation type important for spider monkeys, because the total HR polygons might contain areas that, although used only occasionally, might be crucial for supplying resources in times of scarcity. The mosaic of vegetation found in Punta Laguna is common in the humid tropics, and the heterogenous use of different vegetation types could imply that some of the published figures of the minimum area required for conservation of a viable population (e.g. [57], [58]) could actually be underestimations.

In conclusion, our long-term study confirms the view that spider monkeys show a high degree of site fidelity in their space use patterns. We found that HRs and CAs, while variable across years, consistently included the same areas, which had a higher proportion of high, mature vegetation and that presumably provided them with more reliable and nutritious food. HRs varied more than CAs due to the inclusion of large areas used only sporadically. The method we have used for quantifying site fidelity, consisting of overlaying yearly HR or CA data and comparing these intersections to the total sum of yearly layers, could be of help in studies of other species for which long-term data exist.

## Supporting Information

Figure S1 **Maps of the HR (lighter tones) and CA (darker tones) polygons for each year of study.** Polygons for the W group are shown in green and those for the E group in orange. The thin blue line shows the contour of the Punta Laguna lake. Panel A shows the results of the LoCoH method and Panel B those of the KDE method. Points correspond to the location of all observations with which the polygons were estimated (filled circles: W group; circles with a plus sign: E group). It is important to note that many points contain repeated observations, especially in areas close to the lake. Both the LoCoH and the KDE methods use these repetitions as an indication of intensity of use, giving more weight to those points with the largest numbers of repetitions. The points lying outside of the polygons thus defined have small numbers of repetitions and perhaps constitute occasional explorations in search of new resource areas. As can be seen in Panel B, the KDE method tended to include areas of swamp grassland close to the lake, even though there were no observation points in those areas. Also, the KDE polygons included large areas to the south and southwest of the HR of the W group, whereas the LoCoH estimations did not. As mentioned by Kie et al. [40], the KDE method is particularly sensitive to the bandwidth value used, and commonly overestimates the UDs with excess space around the outmost points. In contrast, by virtue of establishing limits at the actual location data and then estimating the probability of use in the direction of other location data, the LoCoH method establishes more realistic boundaries to the HRs.
(TIF)
