# Supplementary figures and images for: Site Fidelity in Space Use by Spider Monkeys (Ateles geoffroyi) in the Yucatan Peninsula, Mexico
Source: PLoS One. 2013 May 13;8(5):e62813. doi: 10.1371/journal.pone.0062813 (PMC3652831; doi:10.1371/journal.pone.0062813)

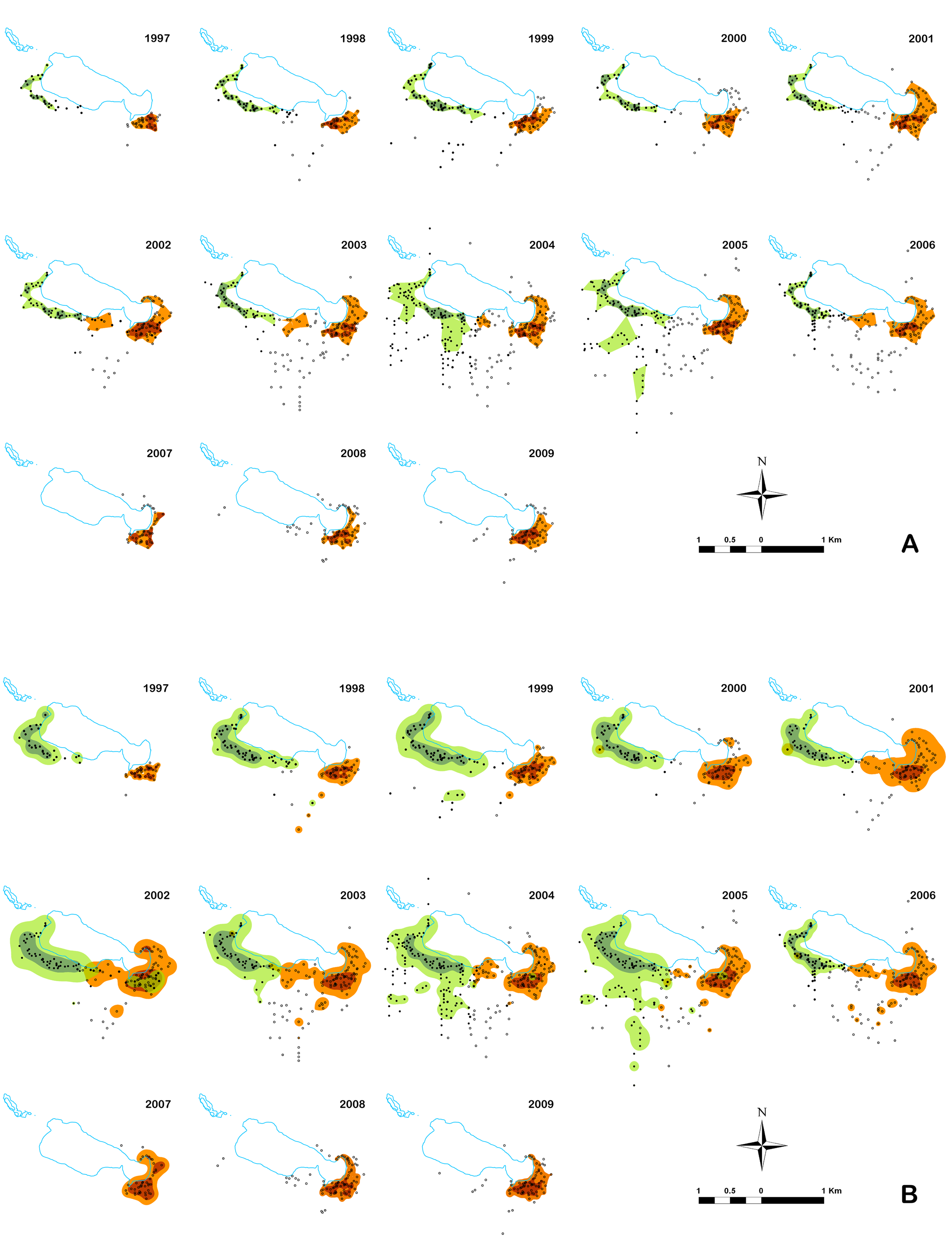

Supplement: Figure S1 — Maps of the HR (lighter tones) and CA (darker tones) polygons for each year of study. Polygons for the W group are shown in green and those for the E group in orange. The thin blue line shows the contour of the Punta Laguna lake. Panel A shows the results of the LoCoH method and Panel B those of the KDE method. Points correspond to the location of all observations with which the polygons were estimated (filled circles: W group; circles with a plus sign: E group). It is important to note that many points contain repeated observations, especially in areas close to the lake. Both the LoCoH and the KDE methods use these repetitions as an indication of intensity of use, giving more weight to those points with the largest numbers of repetitions. The points lying outside of the polygons thus defined have small numbers of repetitions and perhaps constitute occasional explorations in search of new resource areas. As can be seen in Panel B, the KDE method tended to include areas of swamp grassland close to the lake, even though there were no observation points in those areas. Also, the KDE polygons included large areas to the south and southwest of the HR of the W group, whereas the LoCoH estimations did not. As mentioned by Kie et al. [40], the KDE method is particularly sensitive to the bandwidth value used, and commonly overestimates the UDs with excess space around the outmost points. In contrast, by virtue of establishing limits at the actual location data and then estimating the probability of use in the direction of other location data, the LoCoH method establishes more realistic boundaries to the HRs. (TIF) [file pone.0062813.s001.tif]
